# Do All Children with Congenital Complete Atrioventricular Block Require Permanent Pacing ?

**Published:** 2003-07-01

**Authors:** Christian Balmer, Urs Bauersfeld

**Affiliations:** Division of Cardiology University Children's Hospital Zurich, Switzerland

**Keywords:** heart block, congenital, pacemaker, artificial, lupus erythematosus

With an incidence of 1 in 20,000 live born infants [1], congenital complete atrioventricular block (CCAVB) is a rare disease. The aetiology is not completely understood. However, CCAVB may be isolated or combined with congenital heart diseases in up to 53% of affected individuals [2]. Isolated CCAVB is in up to 98% of the children associated with positive autoimmune antibodies in the maternal serum (anti-Ro/SS-A and anti-LA/SS-B) [3,4]. Interestingly, these antibodies are not specifically directed against the conduction system but also against normal myocardial cells and may cause myocarditis [5,6]. Affection of the conduction system can occur at different levels [7]. Histologically, the atrioventricular node tissue may be replaced by fibrous fatty tissue with variable involvement of the distal conduction system [8].

The onset of clinical symptoms in patients with CCAVB is already antenatally in up to 28% [9], but can also occur only later in life. This is due to a variable degree of heart block and heart rate. Most of the symptoms are related to the slow heart rate: hydrops foetalis, heart failure of the neonate, exercise intolerance of the child. Longer pauses may cause praesyncope, syncope (classical Adams Stokes attacks) or even sudden cardiac death. Whether or not Cardiomegaly is mainly the result of a chronic compensatory increased stroke volume secondary to the slow heart rate is somewhat controversial [10]. Cardiomegaly may also be a distinct disease in a subgroup of patients because it does not necessarily resolve with pacemaker (PM) therapy [11,12]. Morbidity and mortality of CCAVB do not seem to correlate with antibody status or associated cardiac lesions [13,14]. There are case reports, that fetal CCAVB can be improved with steroids [15]. Rarely, CCAVB resolves spontaneously [16-19]. In most patients, the degree of conduction abnormality will either persist or worsen over time.

There is no causal therapy for CCAVB. Permanent pacing is the only possible therapy. The aim of PM therapy is to prevent sudden cardiac death and to restore an adequate heart rate to avoid any secondary effects of longstanding bradycardia.

In our clinical practice, the ACC/AHA/NASPE guidelines for PM implantation proved to be useful [20]. Although one has to keep in mind that because of the rareness of the disease and the lack of large randomized trials, the level of evidence is usually low and most of these recommendations are based on experts opinions only.

The following patients with CCAVB should undergo permanent pacing (1): First, all patients with advanced second- or third- degree AV block with symptomatic bradycardia, exercise intolerance or low cardiac output. In children, symptoms can be subtle. In young children there may be poor growth and development, sleep disturbances including night - terror and bed wetting, frequent naps compared to peers and preference for a more sedentary behavior. School children may show poor school performance secondary to tiredness, the need to nap or to go to bed very early after school, irritability, and inability to keep up with peers during exercise [13]. Patients and parents tend to accept and adapt to minor symptoms and recognize them only in retrospect. Often, restored normal heart rates and the chronotropic competence after PM implantation lead to an exciting and motivating gain in energy and perseverance, which can change the personality of the child from lazy and lethargic to energetic and quick.

Second, patients with ECG changes believed to be associated with an increased risk for life threatening arrhythmias such as an unstable escape rhythm, sinus tachycardia, wide QRS escape rhythm, prolonged QT interval or ventricular arrhythmia [20]. The level of evidence is best for the latter two [19,21]. Long RR pauses are acceptable up to 3 seconds while awake or 5 seconds while sleeping [13]. Ambulatory ECG monitoring with frequent episodes of junctional exit block, flat junctional response or associated tachyarrhythmias have been noted in patients with adverse outcomes [22] but are not generally accepted as risk factors for sudden death.

Third, patients with cardiomegaly [11,12,23] or ventricular dysfunction [20]. In some patients, cardiomegaly is part of a dilated cardiomyopathy which can still progress in spite of PM therapy [11,12]. In most patients a decrease in left ventricular size and an improved myocardial function has been demonstrated after PM implantation [23].

The lower limits of an acceptable escape rhythm might be around 55 bpm in neonates and 50 bpm in patients older than one year with the patient being awake and at rest [20]. In a prospective study of 27 patients with CCAVB and a mean age of 6.8 years 8 of 13 patients with mean daytime rates below 50 bpm in ambulatory ECG monitoring had cardiac complications such as sudden cardiac death (n=3), syncope, praesyncope or excessive fatigue [22].

The indication for a PM implantation is also given in patients with CCAVB and significant congenital heart diseases (i.e. those with cyanotic heart disease, single ventricle physiology, complete AVSD or significant AV valve disease) [13]. Again, this recommendation is not strictly evidence based but in these children, the haemodynamic status is often jeopardized from many reasons and restoring a normal heart rate and AV synchrony is often followed by a general improvement and well being.

Unfortunately, a large number of patients with CCAVB do not fall in the categories mentioned above and need an individual assessment. In most of these asymptomatic patients without specific changes in ECG or echocardiography we would still follow a permanent pacing strategy. We have to balance carefully the risks and disadvantages of a PM implantation against the risk of sudden cardiac death. The acceptance of a permanent PM by patients as well as parents is usually extremely good. There are however some specific age and size related particularities.

We try to achieve a haemodynamic situation as close as possible to the normal heart rhythm. Therefore we tend to implant dual chamber PM in most of the children. The miniaturizing of pulse generators nowadays allows for implanting dual chamber pacing systems in babies down to 1600g [24]. In infants and small children we do have very good experiences with bipolar steroid-eluting epicardial pacing leads, attached to the right atrium and right ventricle by a median sternotomy (2) or fixed to the left atrium and left ventricle after a lateral thoracotomy. Since all of the children will need their PM for a lifetime, we push the age limit for transvenous pacing system towards preschool age in order to avoid intimal trauma and obstruction of large veins in order to preserve them for later PM - systems. It is necessary to leave enough length to the leads to allow for growth of the patient. We prefer devices with automatic threshold measurement and output adjustment (AutoCapture) to increase patient safety and to extend device service life since most of the patients will be paced continuously at relatively high rates [25]. Although some children are still asymptomatic during childhood most of the patients with CCAVB will have PM therapy when reaching adulthood [19].

Patients with CCAVB without PM do need a close, at least yearly, follow-up with 12 lead ECG, 24 hour Holter monitoring and echocardiography. Despite the absence of symptoms in childhood, 50% of the patients will develop symptoms in adulthood and 10% will die prematurely [17-19]. With regards to the ECG it is important to know that not only the AV conduction can worsen over time but also prolonged QTc interval can appear the first time in adulthood in patients who previously had normal QTc [17,19].

Patients with CCAVB may present with similar symptoms but in fact it is a mixed group of diseases with a variety of yet not clearly defined aetiologies. Therefore it is very difficult to predict the natural history. There is a lack of evidence with regards to risk factors predicting sudden cardiac death or syncope in patients with CCAVB. With newer PM technologies the indications for PM therapy in children have been extended over the last years so that patients without symptoms and without specific signs in ECG or echocardiography may also benefit from the advantages of a permanent PM.

## Figures and Tables

**Figure 1:**
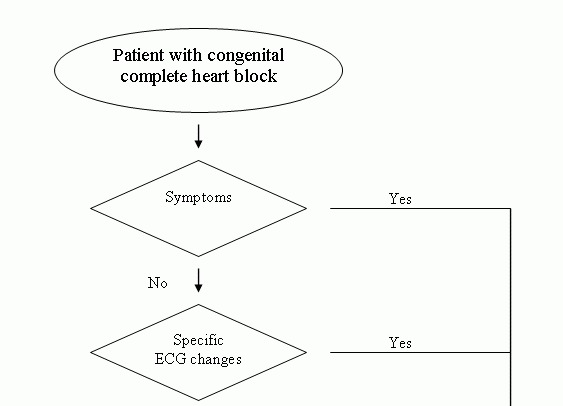

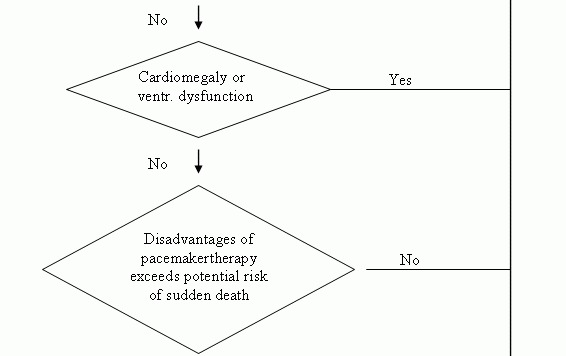

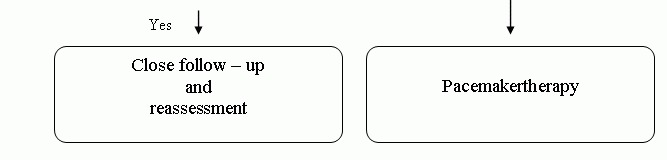
Management of Patients with congenital compete heart block. For details see text.

**Figure 2:**
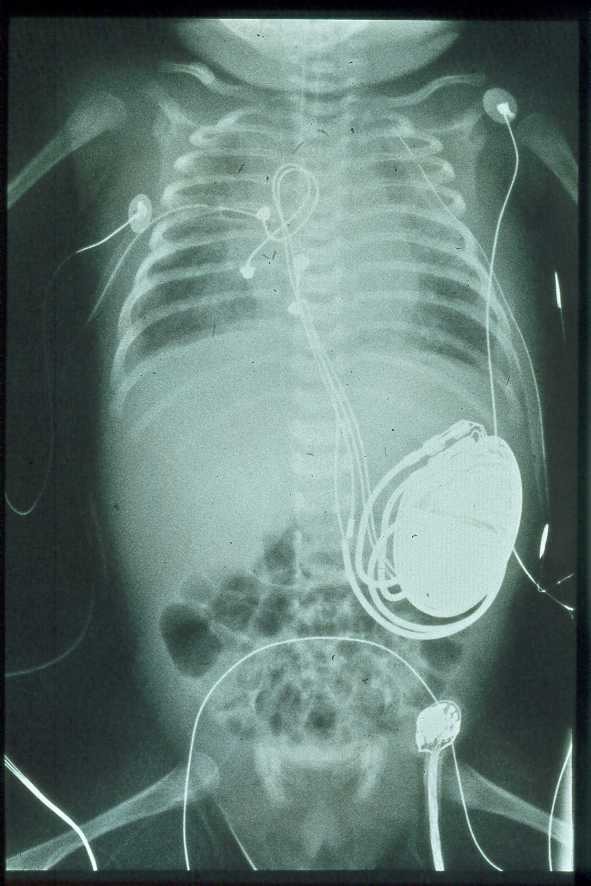
Dual chamber pacemaker with epicardial electrodes implanted through a median sternotomy in a newborn with complete heart block.
